# 60404 HIV Tat Induced Neuroinflammation

**DOI:** 10.1017/cts.2021.410

**Published:** 2021-03-30

**Authors:** Sean Carey, Kathleen Maguire-Zeiss

**Affiliations:** 1 Georgetown University, Department of Biology; 2 Georgetown University Medical Center, Department of Neuroscience

## Abstract

ABSTRACT IMPACT: Demonstrate the role of astrocyte released MMPs in response to pathogenic HIV protein Tat. OBJECTIVES/GOALS: In the presence of the pathogenic HIV protein Tat, astrocytes have been demonstrated to adopt an inflammatory phenotype as well as release extracellular matrix degrading enzymes, MMPs. Our work aims to identify whether MMPs alter perineuronal net integrity and working memory in a mouse model of Tat-induced neuroinflammation. METHODS/STUDY POPULATION: Stereotaxic Injection: C57BL6/J mice were injected bilaterally with HIV-1 IIIB Tat 5ug in 5uL or Vehicle (0.2M KCl, 5mM DTT, 50mM Tris, pH 8.0), into the hippocampus (CA1; -1.9mm AP, ±1.6mm ML, -1.5mm DV from pial surface). All outcome measurements were performed 14-days post injection. Behavior: T-maze was used to assess working memory following Tat exposure. qRT-PCR: TaqMan probes were used according to manufacturer on extracted whole hippocampus mRNA. IF: GFAP and CD68 immunofluorescence was used to determine inflammation post injection. Inhibitory interneurons (parvalbumin positive) and peri-neuronal nets (WFA positive) were quantified. WB: Synaptosomes from whole hippocampi (Syn-PER) were isolated and synaptic excitatory markers were quantified (PSD-95, synaptophysin, GluR2a). RESULTS/ANTICIPATED RESULTS: Tat exposure resulted in impairments in working memory as measured by T-maze alternations and an increase in hippocampal mRNA expression of MMP-13 and IL-1β, indicative of neuroinflammation. We also noted an increase in GFAP+ injection site width 14 days post-Tat injection, suggesting robust gliosis. While there were no changes in the excitatory pre and post synaptic markers we found a significant decrease in the percent of PV+ interneurons with peri-neuronal nets (PNNs) following Tat exposure. Taken together, this preliminary data supports a role for inflammation and PNN integrity in Tat-induced alterations in working memory. DISCUSSION/SIGNIFICANCE OF FINDINGS: Our findings suggest that Tat contributes to cognitive impairment and that astrogliosis with elevated MMP-13 facilitates the degradation of peri-neuronal nets (PNNs) within the hippocampus. Since PNN degradation can alter neuronal circuitry future studies will focus on Tat-induced changes in hippocampal signaling.

